# Prenatal diagnosis of Noonan syndrome in a set of monozygotic twins- a case report

**DOI:** 10.1186/s12884-022-05323-5

**Published:** 2023-01-06

**Authors:** Wei Jian, Huizhen Yuan, Yu Liu, Jimei Sun, Fei Chen, Yufan Li, Min Chen

**Affiliations:** 1 Department of Obstetrics and Gynecology, Department of Fetal Medicine and Prenatal Diagnosis, Guangdong Provincial Key Laboratory of Major Obstetric Diseases, The Third Affiliated Hospital of Guangzhou Medical University, Guangzhou, China; 2 Jiangxi Key Laboratory of Birth Defect Prevention and Control, Jiangxi Maternal and Child Health Hospital, Guangzhou, China

**Keywords:** Noonan syndrome, Cystic hygroma, LZTR1 gene, Prenatal diagnosis, Twin pregnancy

## Abstract

**Background:**

We report a pair of dichorionic diamniotic (DCDA) twin pregnancy affected by Noonan syndrome (NS) with a novel mutation of LZTR1 determined by genetic analysis.

**Case presentation:**

A pregnant woman with monozygotic twins (DCDA) at 12 + 2 weeks gestation was referred to our center. This was her second pregnancy following a previous delivery of a healthy infant. Nuchal translucency of two fetuses was 11.2 mm (CRL 62.0 mm) and 6.9 mm (CRL 62.1 mm) respectively. Ultrasound examination indicated cystic hygroma and hypoplastic ear. The couple was not consanguineous, and both had normal phenotype. Familial hereditary disease was also excluded. Under ultrasound guidance, 30 mg of chorionic villi was obtained for karyotyping, quantitative fluorescent polymerase chain reaction (QF-PCR), chromosomal microarray analysis(CMA), and Trio-whole-exome sequencing(WES) examination. We used the “target region capture and sequencing” for WES, and the BWA (Burrows Wheeler Aligner) Multi-Vision software package for the data analysis. The results of all these tests were normal except WES detected a c.427 A > G mutation in the exonic region of the LZTR1 gene and a p. Asn143Asp novel heterozygous mutation associated with NS in this pair of twins. In addition, WES suggested that the mutation in the twin fetuses originated from the mother. When the mother got the genetic test report, she came to our fetal medicine department for genetic counseling and she declined the appointment with a clinical geneticist. The couple opted to terminate the pregnancy. Because the patient did not choose to terminate the pregnancy at our hospital, we were unable to take further examination. With the help of colleagues in another hospital, photos of the fetuses were taken. Compared with the prenatal ultrasound results, the appearance of the “cystic hygroma” and “hypoplastic ear” was consistent with the ultrasound. The couple were depressed after knowing this pathogenic result and although we advised the mother to take further investigation, they refused.

**Conclusion:**

The mutant locus might be incompletely dominant, which led to an abnormal fetal phenotype such as cystic hygroma and hypoplastic ear.

## Background

Cystic hygroma (CH) is a rare benign cystic lymphatic lesion; nearly three-quarters of CH cases occur in the head and neck. The incidence of CH is estimated to be 1 case per 1000–6000 live births [1]. Approximately 50% of CH cases are caused by a chromosomal aneuploidy, such as trisomy 21/13/18 and Turner syndrome. Noonan syndrome (NS, OMIM number:163,950) is the major cause of CH with single gene disorder [2]. Most cases are sporadic, but can be inherited in an autosomal-dominant manner, affecting males and females equally. This syndrome is characterized by dysmorphic facies, short stature, webbed neck, lymphatic abnormalities, skeletal deformities, bleeding, diatheses, renal diseases, variable intellectual disabilities, and a group of cardiac anomalies [3, 4]. Prenatal ultrasound and molecular genetic test have enhanced the prenatal diagnosis of NS. Here, we report a pair of dichorionic diamniotic (DCDA) twin pregnancies affected by NS with a novel mutation of LZTR1 detected by ultrasonography and WES analysis.

## Case presentation

**Fig. 1:**
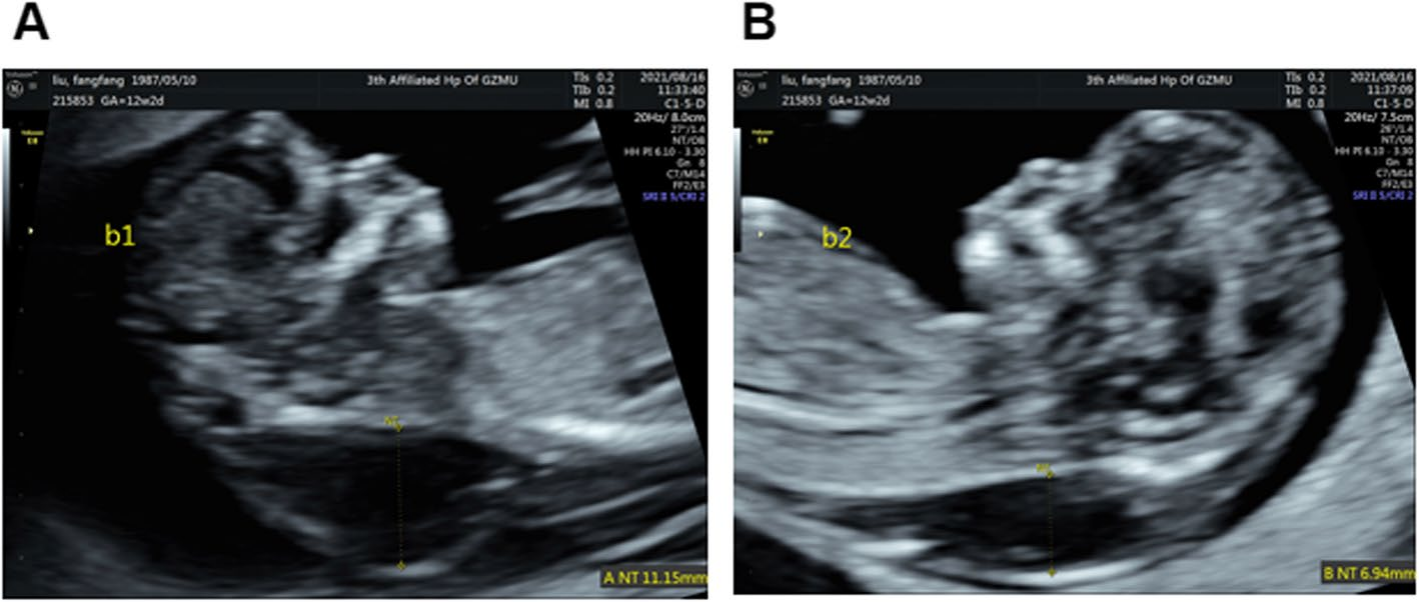
Ultrasonic image of twin fetal NT. **A **Ultrasonic image of fetal 1 NT. **B **Ultrasonic image of fetal 2 NT

**Fig. 2:**
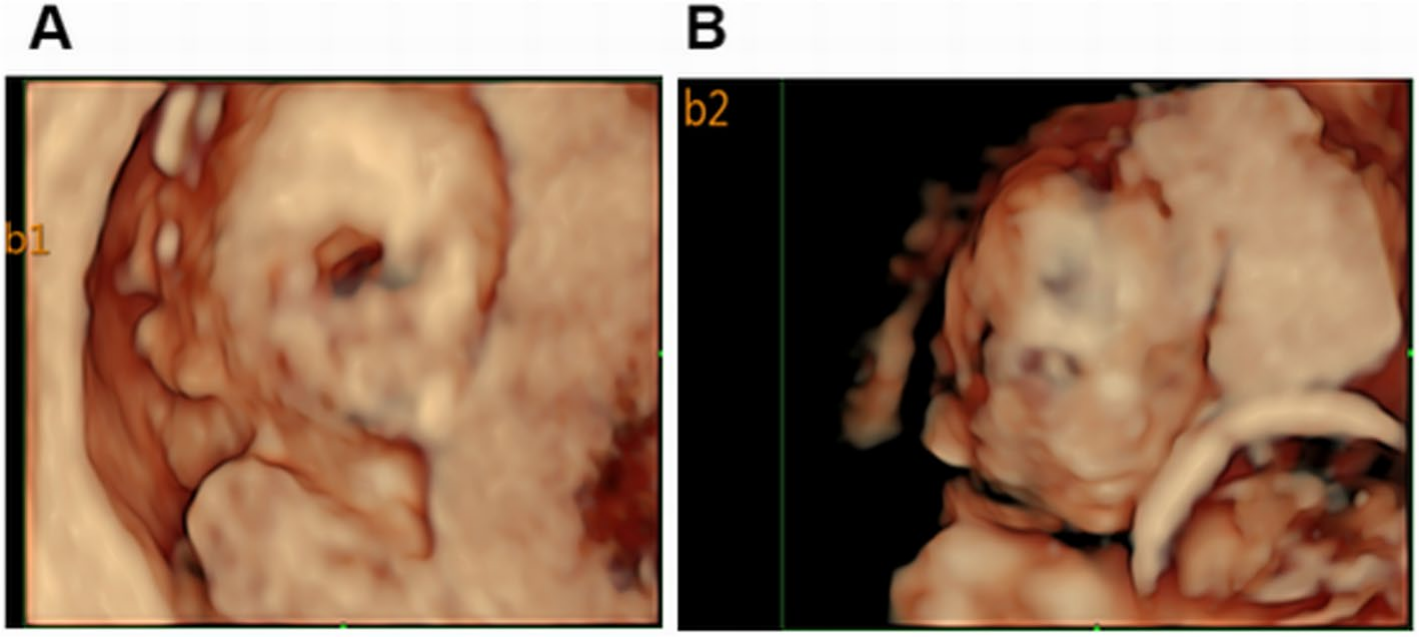
3D images of twin fetuses. **A **3D image of fetus 1. **B **3D image of fetus 2

**Fig. 3:**
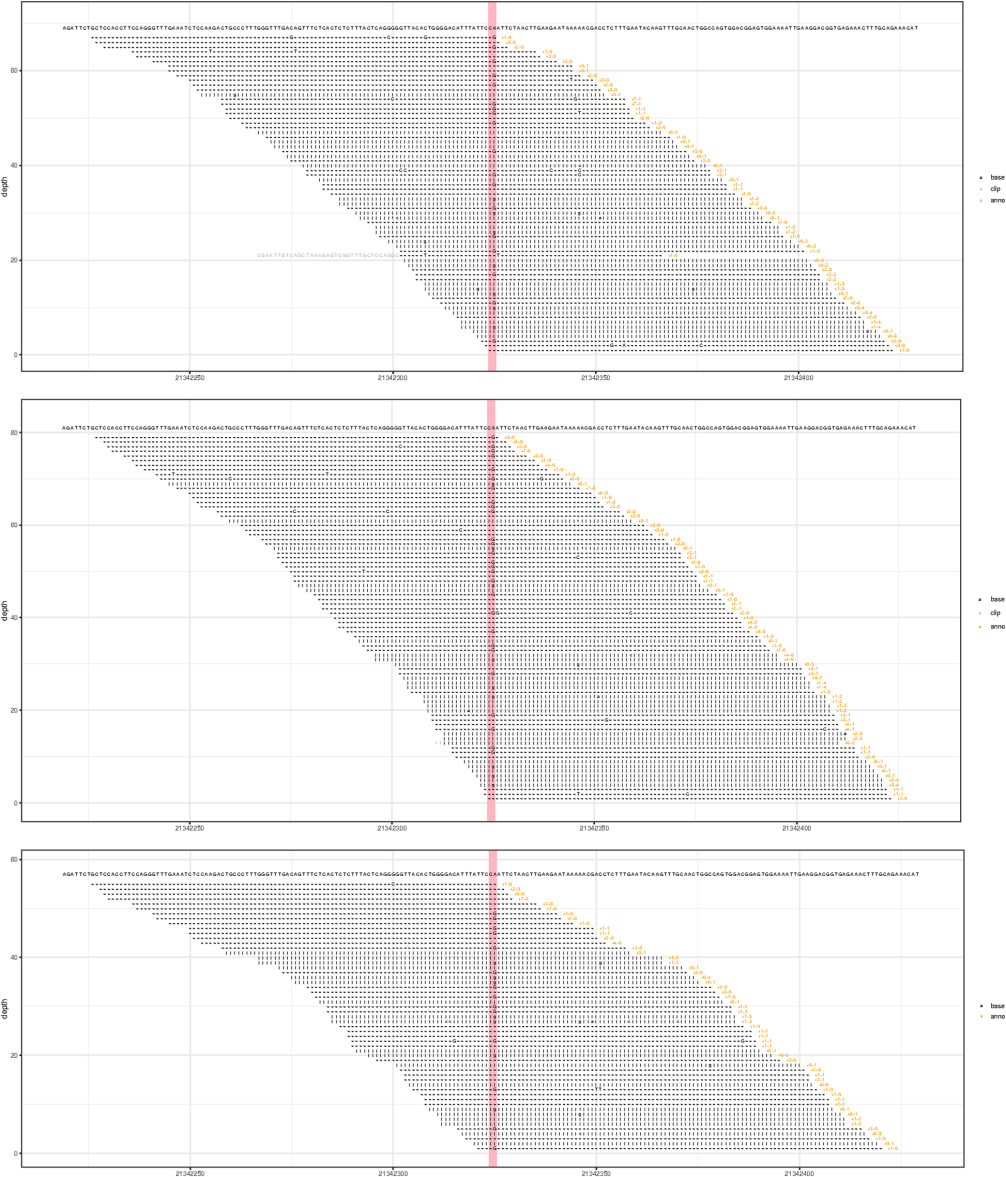
WES identifies an identical heterozygous mutation in the LZTR1 gene (NM_006767.3:c.427 A > G(p.Asnl43Asp)), corresponding to the twin fetuses and the mother chr22:2134–2325. From top to bottom, fetus 1, mother, and fetus 2 are presented, respectively

**Fig. 4:**
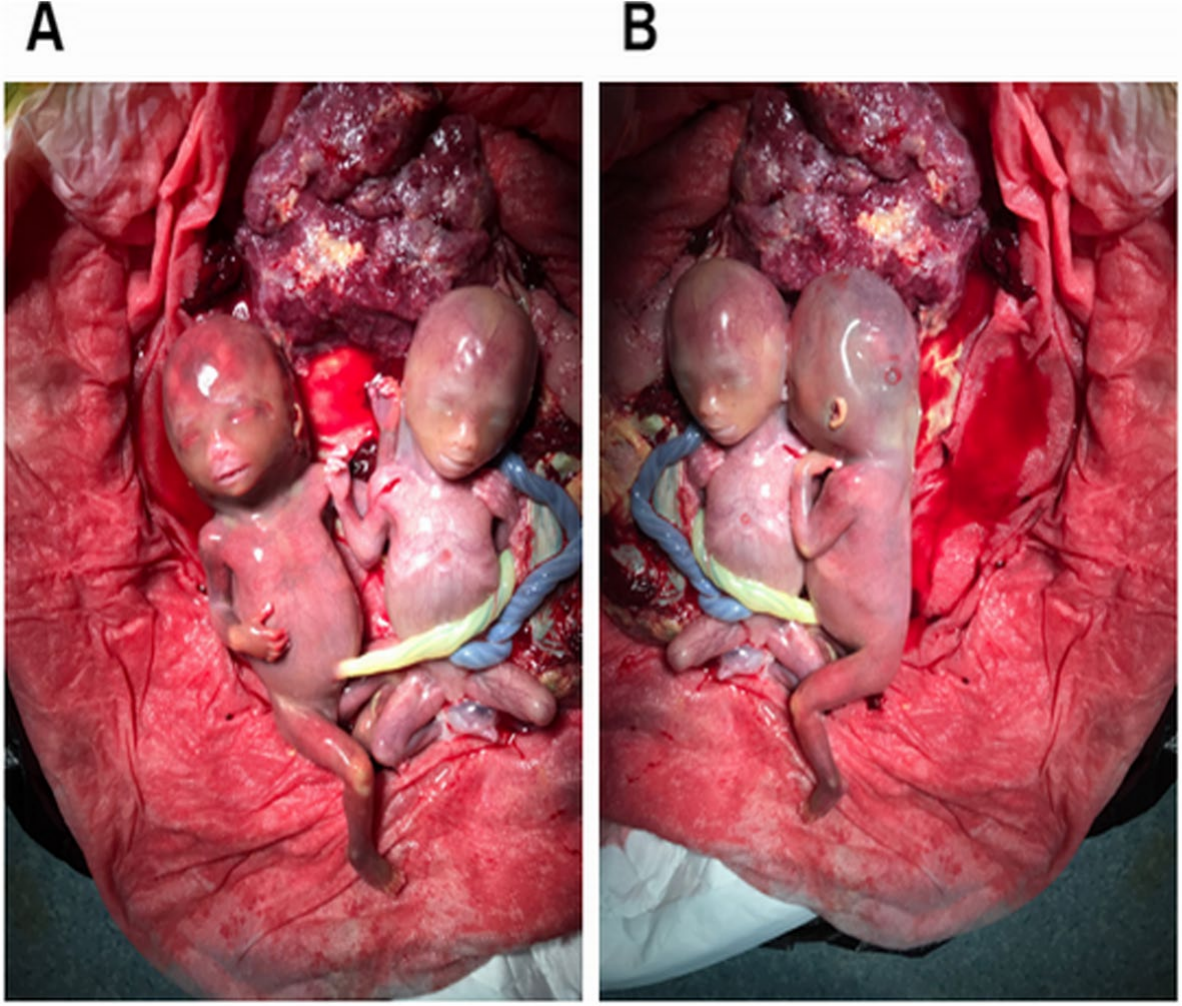
Images of the twin fetuses after termination of pregnancy

A pregnant woman with monozygotic twins (DCDA) at 12 + 2weeks gestation was referred to our center. This was her second pregnancy following a previous delivery of a healthy infant. Nuchal translucency (NT) of two fetuses was 11.2 mm (CRL 62.0 mm) and 6.9 mm (CRL 62.1 mm), respectively. Ultrasound examination indicated CH and hypoplastic ear as shown in Figs. 1 and 2. The couple was not consanguineous. Their phenotype was normal, and the possibility of familial hereditary disease was also excluded. Under the guidance of ultrasound, 30 mg of fetal chorionic villus was obtained for karyotyping, QF-PCR, CMA, and Trio-WES examination. We used the ***“*** target region capture and sequencing” for WES, and the BWA (Burrows Wheeler Aligner) Multi-Vision software package for the data analysis. The results of all these tests were normal; however, WES detected a c.427 A > G mutation in the exonic region of the LZTR1 gene and a p.Asn143Asp novel heterozygous mutation associated with NS in this pair of twins. In addition, WES suggested that the mutation in twin fetuses originated from the mother (Fig. 3). When the mother got the genetic test report, she came to our fetal medicine department for genetic counseling and she declined the appointment with a clinical geneticist. Ultimately, this family chose to terminate the pregnancy (Fig. 4). Because the patient did not choose to terminate the pregnancy at our hospital, we were unable to take further examination. With the help of colleagues, photos were taken of the fetus. Compared with the prenatal ultrasound results, the appearance of the “cystic hygroma” and “hypoplastic ear” was consistent with the ultrasound. The couple were depressed after knowing this pathogenic result and although we advised the mother to take further investigation, they refused.

## Discussion and conclusions

NT is the thickness of fluid collection in the fetal neck, which can be observed by an ultrasound scan between 11 and 13 + 6 weeks of gestation. Increased NT has been reported to be associated with chromosomal abnormalities and fetal structural defects [5, 6]. CH can be clearly differentiated from a simple increase in NT. The typical ultrasonographic appearance of CH is that of two dilated jugular lymphatic sacs seen on a transverse scan at the level of the neck and separate from the NT. In fetuses with CH and normal karyotype, at least 9-16% were associated with NS [7]. In the current study, the results of karyotype, QF-PCR and CMA examination were all normal. WES identified a c.427 A > G heterozygous mutation in the exonic region of the LZTR1 gene, which was confirmed to be maternal origin. It was associated with NS type 10 (autosomal dominant, AD), nerve sheath tumor disease type 2 susceptibility, (AD), and NS type 2 (autosomal recessive) [8]. NS is known as an AD disease that is characterized by short stature, craniofacial dysmorphism, short or webbed neck, cardiac anomalies, cryptorchidism, and coagulation defects [9]. NS is associated with variations of genes regulated by the RAS/MAPK signaling pathway. PTPN11 contributes to 50% of the mutant gene, followed by SOS1 (10-15%), RAF1 (5-15%), TIT1 (4-9%), KRAS (< 5%), NRAS (< 1%), BRAF (< 1%), SHOC2 (< 1%), SPRED1 (< 1%), MEK1 (< 1%), and CBL (< 1%) [10, 11]. All these genes account for the clinical diagnosis of 80% of NS. Yamamoto et al. [12] identified two novel genes (SOS2 and LZTR1) that may lead to NS, and greatly expanded the genetic spectrum of RAS. They identified eight LZTR1 gene mutants associated with NS in seven patients, including a patient with variants in both alleles, and all missense variants that detected were predicted to be deleterious by software. The results suggested that LZTR1 is the causative gene for AD or ARNS and that LZTR1 interacts with the RAF1/SHOC2/PPP1CB complex and regulates the phosphorylation of Ser259 on RAF1, which leads to the clinical manifestations of NS [13]. Defining the episodic rate of NS for mild phenotype in adults is challenging; some patients remain undiagnosed until the birth of their pathogenic infants. We confirmed that the genetic variants in fetuses were originated from the mother with normal phenotypes; however, we could not determine whether the gross normal mother was associated with a mild phenotype or incomplete penetration. The two mutant loci c.427 A > G and p.Asn143Asp located in Kelch domain (positions 79–285) are extremely rare, and they are predicted to be deleterious by software. Most dominant variants occur in this protein functional domain [14], which upregulate the stress-dependent MAPK signaling pathway and thus result in disease [15]. In summary, LZTR1-associated NS in the two fetuses exhibits similar clinical phenotype to normal karyotype such as increased NT and CH. The c.427 A > G and p.Asn143Asp variant identified in the fetuses of this study is extremely rare and highly pathogenic. Unfortunately, we could not perform genetic co-segregation analysis due to the absence of samples from other family members. More evidence is needed to confirm the pathogenicity of the mutant. Further study shall continue collecting cases with LZTR1 mutation and conduct functional studies to further clarify the association of the LZTR1 gene with NS.

## Data Availability

The datasets generated during the current study are available from the corresponding author on reasonable request. The authors may not be able to make their datasets publicly available to protect the patients’ privacy.

## Abbreviations

NS: Noonan syndrome
DCDA: Dichorionic diamniotic
CH: Cystic hygroma
NT: Nuchal translucency
AD: Autosomal dominant
QF-PCR: Quantitative fluorescent polymerase chain reaction
CMA: Chromosomal microarray analysis
WES: Whole-exome sequencing
